# A call to eradicate non-inclusive terms from the life sciences

**DOI:** 10.7554/eLife.65604

**Published:** 2021-02-08

**Authors:** Aziz Khan

**Affiliations:** Stanford Cancer Institute, School of Medicine, Stanford UniversityStanfordUnited States; eLifeUnited Kingdom; eLifeUnited Kingdom

**Keywords:** equity, diversity, inclusion, systemic racism, scientific language, non-inclusive terms, research culture, None

## Abstract

Since the Black Lives Matter movement rose to mainstream prominence, the academic enterprise has started recognizing the systematic racism present in science. However, there have been relatively few efforts to make sure that the language used to communicate science is inclusive. Here, I quantify the number of research articles published between 2000 and 2020 that contained non-inclusive terms with racial connotations, such as “blacklist” and “whitelist”, or “master” and “slave”. This reveals that non-inclusive language is being increasingly used in the life sciences literature, and I urge the global academic community to expunge these archaic terms to make science inclusive for everyone.

Historically, many terms are associated with racial connotations. In the tech world, the words “master” and “slave” are often used to refer to types of storages, circuits, databases or code, in which the slave type is subservient to the master. Other commonly used terms are “blacklist” and “whitelist” — where the blacklists are the problematic entities and whitelists are the good ones (Alter et al., 2016).

These, and several other archaic and non-inclusive terms, are also widely used in scientific manuscripts (Baeckens et al., 2020; Herbers, 2007; Houghton and Houghton, 2018). In publishing, the term “blacklist” is used to filter out predatory journals and publishers from non-predatory and more trustworthy journals that are added to the “whitelist” (Houghton and Houghton, 2018; Silver, 2017). In the life sciences, the term “blacklist” is commonly used to represent problematic genomic regions, variations, genes, or proteins which need to be filtered out as an artifact or noise (Wimberley and Heber, 2019; Maffucci et al., 2019; Collins et al., 2019; Wilfert et al., 2016). For example, the ENCODE blacklist regions are a curated list of non-coding regions in the genome, which is used by the gene regulation community – including myself – as an essential quality filter when analyzing genomic and epigenomic data (Amemiya et al., 2019).

The terms “master” and “slave” are also frequently used in molecular biology to group transcription factors (TFs) or genes based on their function. For example, proteins that are at the top of the regulatory hierarchy and control key biological programs, such as determining a cell’s fate, are commonly named “master regulators” or “master TFs”. While some may argue that it is acceptable to use the term “master”, the problem gets worse when some researchers introduce "slave TFs" (Ocone and Sanguinetti, 2011).

## Use of non-inclusive terms in life sciences literature is growing

**Figure 1.**
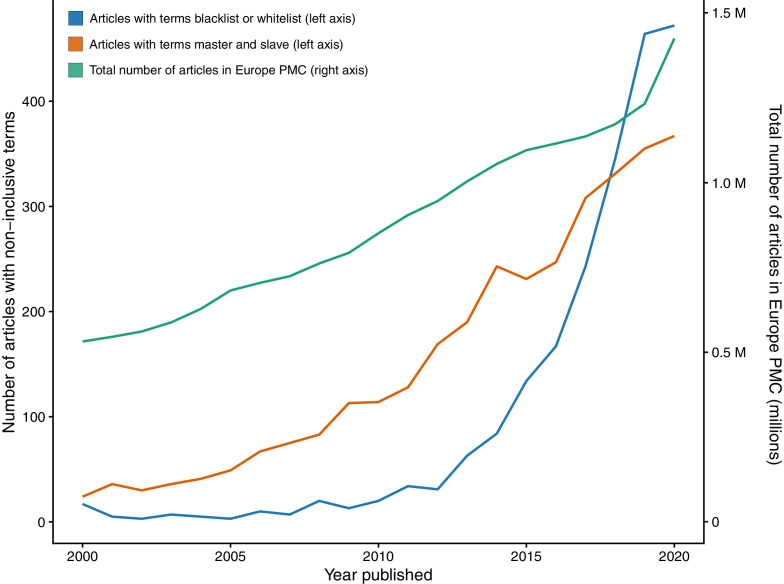
The growth of non-inclusive terms in the life sciences literature. The number of articles on Europe PMC containing the terms blacklist or whitelist (blue; left axis), containing the terms master and slave (orange; left axis), and the total number of articles on Europe PMC (green; right axis) between 2020 and 2000.

To estimate the use of the terms blacklist/whitelist and master/slave, I performed searches on the open-access repository Europe PMC which contains millions of biomedical research articles. A search for articles containing blacklist/whitelist returned more than 2,000 articles published in more than 600 journals between 2000 and 2020 (Figure 1), with blacklist appearing more often (1,994 articles) than whitelist (439 articles).

**Figure 1—figure supplement 1.**
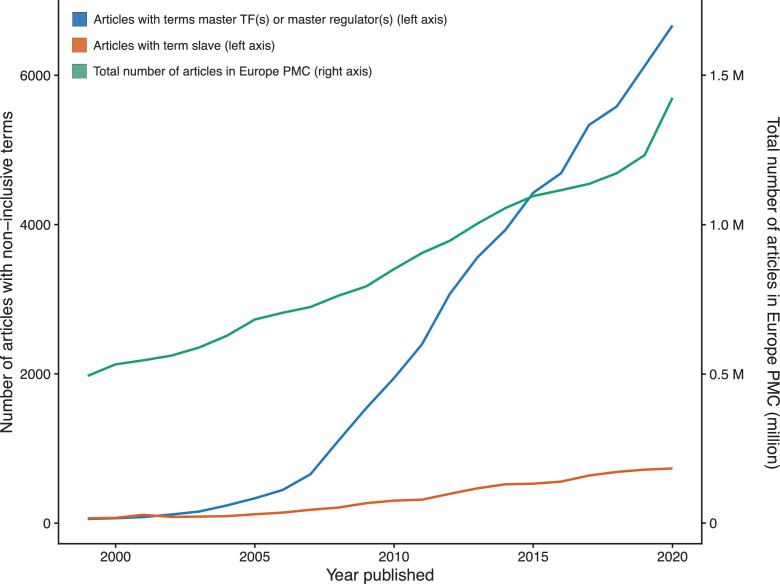
The number articles in the life sciences literature that just contain the term "master" or "slave". The number of articles on Europe PMC containing the terms master TF(s) or master regulator(s) (blue; left axis), containing the term slave (orange; left axis), and the total number of articles on Europe PMC (green; right axis) between 2020 and 2000.

The first use of the term “blacklist” dates back to the seventeenth century and has a long history of being used in the labor market (Weir, 2013). However, these terms started appearing in the biomedical literature around the mid-nineteenth century. In 1899, an article in the journal The Hospital suggested maintaining a “whitelist” of firms that treat their employees fairly instead of a “blacklist" of firms with a bad reputation (The Hospital, 1899). Since then, the use of these non-inclusive terms has continued to grow (Figure 1).

The terms “master” and “slave” are also widely used in the scientific literature. A search for articles with both these terms found over 3,500 research articles published in more than 900 journals between 2000 and 2020 (Figure 1). Similar to blacklist and whitelist, the use of master and slave is growing with time. Furthermore, a search for “master TFs” or “master regulators” found more than 50,000 articles from 2000 to 2020, with their use increasing each year (Figure 1—figure supplement 1). This suggests that non-inclusive terms are becoming increasingly pervasive, and possibly the norm in the life sciences literature.

Most of the papers with non-inclusive terms were published in well-known journals, including multidisciplinary journals (such as Nature, Nature Communications, PLOS One, PNAS and Scientific Reports) and journals with broad scopes within the life sciences and medicine (such as BMJ, Cell, Cell Reports and eLife). In addition to these multidisciplinary and broad-scope journals, the journals that used the terms "blacklist" or "whitelist" most often were BMC Bioinformatics, Nature Genetics and Genome Research, and the journals that used the terms "master" and "slave" most often were Sensors, Optics Express, Scientific World Journal and BMC Bioinformatics. Inevitably, larger journals (such as Nature Communications, PLOS One, PNAS, Scientific Reports and Sensors) tended to use these terms more often than small journals with fewer publications.

## Let’s expunge non-inclusive terms to make science inclusive for all

Following the Black Lives Matter protests the scientific community has spoken against the systematic racism in science and called for action to make science more diverse and inclusive (Barber et al., 2020; Cell Editorial Team, 2020; Eisen, 2020; Nature, 2020; Sanford, 2020; Stevens et al., 2021; Taffe and Gilpin, 2021). Yet, the growing use of such non-inclusive terms in scientific literature potentially reflects a racist research space that endorses and sustains the use of these terms. The more we use this language, the more it becomes a habit, and we need to act now to avoid passing this behavior on to future generations of scientists.

Some tech and governmental organizations, such as Google, GitHub, the UK National Cyber Security Center, among others Seele, 2020, are already replacing such terms that reflect a racist culture (Google, 2020; GitHub, 2020; Emma, 2020; Seele, 2020; Im, 2020). I urge the scientific community (including institutions, researchers, funders, learned societies, journals and others) to follow suit, and replace the terms blacklist/whitelist with excluded/included or deny/allow lists, and to use the terms primary and secondary instead of master and slave.

There are several other examples of non-inclusive terminologies that are used in the life sciences and beyond. For example, there are growing concerns over terms with racial etymology, such as “slave-making ants” — a slavery metaphor to describe ant behavior (Herbers, 2020; Herbers, 2007), or the word “noosing” to describe catching lizards, which reminds people of the racial lynchings of Black people in the United States (Cahan, 2020). A number of plant and animal species also have non-inclusive names or are named after people who were known for their racist rhetoric (Shiffman, 2019).

Recently, the racially loaded term “quantum supremacy” was introduced to represent the power of quantum computers, which is now getting replaced by “quantum advantage” (Palacios-Berraquero et al., 2019; Wiesner, 2017). Additionally, in response to recent social unrest, the academic enterprise has started renaming academic buildings, programs and prizes, and removing monuments named after people who were known for their racist comments and ideology (Cahan, 2020). Now, it is time for us to also rethink the language we use to communicate science.

Language matters — it shapes the way we think, see and behave. The list of non-inclusive terms in science is long and widespread across multiple disciplines. As scientists, we have a responsibility to fix the problem and to use language that is inclusive to everyone.

## Methods

The research articles with specific terms were queried through Europe PMC using the *europepmc* R package v0.4 (Ferguson et al., 2021). The search query was restricted to publication year between January 01, 2000, to December 31, 2020. Preprints were excluded from the search.

The query used to search articles with terms blacklist and whitelist is as follows: ((blacklist OR blacklisted OR “black-listed” OR “black-list” OR blacklisting) OR (whitelist OR whitelisted OR “white-listed” OR “white-list” OR whitelisting)) AND (FIRST_PDATE:[2000-01-01 TO 2020-12-31]) NOT (SRC:PPR).

The query used to search articles with terms master and slave is as follows: (“master” AND “slave”) AND (FIRST_PDATE:[2000-01-01 TO 2020-12-31]) NOT (SRC:PPR).

The query used to search articles with master TF(s) or master regulator(s) is as follows: ("master TFs" OR "Master transcription factor" OR "master regulator" OR "master TF") AND (FIRST_PDATE:[2000-01-01 TO 2020-12-31]) NOT (SRC:PPR).

All the figures were created using ggplot2 v3.3.2 Wickham, 2016 with R v3.6.1. The figures can be reproduced using the available code in the code and data availability section (Wickham, 2016).

### Code and data availability

The source code and data used to generate figures are available on GitHub (https://github.com/asntech/inclusive-science) and also on Zenodo (Khan, 2021).

## Data Availability

The source code and data used to generate figures are available on GitHub (https://github.com/asntech/inclusive-science; copy archived at https://github.com/asntech/inclusive-science/releases/tag/v1.1) and also on Zenodo (https://doi.org/10.5281/zenodo.4458453). The following dataset was generated: AzizK2021A call to eradicate non-inclusive terms from scienceZenodo10.5281/zenodo.4458453PMC787013733556000

## Additional files

Transparent reporting form
